# Development and psychometric evaluation of TEXP-Q: a questionnaire measuring transition and transfer experiences in emerging adults with type 1 diabetes

**DOI:** 10.1186/s41687-023-00652-1

**Published:** 2023-11-08

**Authors:** Mikaela Vallmark, Anna Lena Brorsson, Carina Sparud-Lundin, Ewa-Lena Bratt, Philip Moons, Markus Saarijärvi, Mariela Acuña Mora

**Affiliations:** 1 Institute of Health and Care Sciences, Sahlgrenska Academy, University of Gothenburg, Gothenburg, Sweden; 2 Department of Neurobiology, Care Sciences and Society, Karolinska Institutet, Stockholm, Sweden; 3 Gothenburg Centre for Person-Centred Care (GPCC) at University of Gothenburg, Gothenburg, Sweden; 4 Region Västra Götaland, Sahlgrenska University Hospital, Children’s Heart Center, Gothenburg, Sweden; 5 Department of Public Health and Primary Care, KU Leuven, Leuven, Belgium; 6 Department of Paediatrics and Child Health, University of Cape Town, Cape Town, South Africa; 7 Institute of Healthcare Sciences, Faculty of Caring Science, Work Life and Social Welfare, University of Borås, Borås, Sweden

**Keywords:** Transition, Type 1 Diabetes, Emerging adults, Questionnaire, Person-centred care, Psychometric evaluation

## Abstract

**Background:**

During transition to adulthood and transfer to adult healthcare, emerging adults with chronic conditions are at risk of deteriorating disease control, well-being, and acute, as well as long-term complications. Despite an increasing call for person-centred healthcare services attuned to young peoples’ needs, few validated instruments exist pinpointing adolescents’ and emerging adults’ experiences of preparation for transition and transfer. Thus, the overarching purpose of this study was to develop a person-centred, clinically applicable instrument (Transitional care EXPeriences Questionnaire, TEXP-Q) adjustable to different chronic conditions, although the focus in the present study was Type 1 Diabetes. The specific aim was, therefore, to describe the development and psychometric evaluation of TEXP-Q in emerging adults with Type 1 Diabetes.

**Methods:**

Initial development of the TEXP-Q was inspired by existing research. Items were formulated in accordance with consensus recommendations for developing patient-reported measures, and extra consideration was taken to ensure person-centredness. Psychometric evaluation comprised two phases: In phase I, data from cognitive interviews, content validity indexing, and judgement of an expert panel provided information on face and content validity. In phase II, data from a cross-sectional study conducted at eight adult diabetes outpatient clinics in Sweden (n = 163) allowed for explorative factor analysis (EFA), as well as calculation of content validity, reliability and responsiveness.

**Results:**

Combining results from cognitive interviews, content validity index values and expert panel judgement, a test version of TEXP-Q was developed, the content and face validity of which were considered good. This version consisted of 17 items answered on a five-point Likert scale, and three open-ended questions answered in free text. During EFA, four items were removed, and a three-factor solution was recognised as most adequate, accounting for 60% cumulative variance and one single cross-loading. After EFA, the instrument comprised 13 questions, divided into three latent factors. Cronbach’s alpha for the complete instrument was 0.866, which indicates good internal consistency. Crohnbach’s alpha approximated to 0.8 for all factors respectively.

**Conclusion:**

TEXP-Q is a newly developed, person-centred instrument which has proven to be both valid and reliable when applied to youths with T1D. The questionnaire fills a need for instruments focusing on emerging adults’ experiences of preparation for transition and transfer.

## Background

Growing up with a chronic condition, such as Type 1 Diabetes (T1D), adds an extra, often multifaceted burden on those affected, with reciprocal consequences on condition and adolescent development [1–3]. As a result, young people with chronic conditions – defined as conditions lasting twelve months or more and resulting in functional limitations and/or a need for ongoing medical care [4] – are often afflicted with deterioration in disease control and well-being, potentially leading to long-term complications [5]. Moreover, the late teens and early twenties constitute the point in time when transfer from paediatric to adult care usually takes place. The concepts *transition* and *transfer* imply different phenomena in their lives, as well as in the scope of healthcare for young people with chronic conditions. While *transfer of care* defines the plain, physical movement from paediatric to adult healthcare services, *transition of care* stands for the complex, continuous shift of focus and responsibility from family and caregivers to the young people themselves [6].

During emerging adulthood, transition readiness, as well as transfer experiences and satisfaction, have emerged as key elements to both measure and attain [7, 8], as have disease-related knowledge, self-management, and follow-up rates [9]. Since the 1950s, the concept of person-centred care (PCC) has gained ground, moving the patient from being a passive receiver of care to being the reference point instead, and participating actively in all stages of his/her treatment and care [10, 11]. In close connection with the appeal for person-centredness in PCC, the use of patient-reported measures is advancing in both research and clinical settings [12, 13]. Whereas patient-reported outcome measures (PROMs) capture patients’ perspectives on treatment outcomes – most often mirrored by self-perceived health status during or after treatment – patient-reported experience measures (PREMs) grasp experiences of (or satisfaction with) the treatment procedure, structure, or outcomes. Although the direction of the relationship is often diffuse and difficult to isolate, numerous studies have proven a positive relationship between patient satisfaction and health outcomes [12, 14]. In contrast, research on components of person-centred care and their potential impact on emerging adults’ transition to adulthood and transfer to adult care is limited, and few validated instruments exist pinpointing young peoples’ experiences prior to, during, and after transfer [1, 8]. Thus, strengths and weaknesses in the delivery of care remain unclear, and by extension, possible adjustments needed to better meet the needs of preparing emerging adults for transition and transfer to adult care. The overarching intention of this study therefore was to develop a person-centred and clinically applicable instrument, subsequently named Transitional care EXPeriences Questionnaire (TEXP-Q), the content and design of which should be adjustable to different chronic conditions, even though the focus in the present study was T1D. Hence, the specific aim of this study was to describe the development and psychometric evaluation of TEXP-Q in emerging adults with T1D.

## Methods

### Scale development

To develop potential items we used literature reviews, qualitative studies and two existing questionnaires in the English language that measure aspects related to transitional care: *Six Core Elements* [15] and *Mind the gap* [16]. Moreover, thorough experience of transitional issues from both clinical practice and research within the research group was guided the process, and resulted in an initial version of TEXP-Q (version 1). Extra care was taken to ensure person-centredness in terms of covering respondents’ ability to share their narrative and to participate in decision-making, treatment, and documentation of their care. Moreover, and in accordance with consensus recommendations for developing patient-reported measures, questions were formulated to cover disease-specific as well as generic phenomena, and pinpoint experiences in favour of global satisfaction with care [12, 13]. Both open-ended and closed questions were included, and for the latter, answers were given on a seven-point Likert scale (“strongly disagree” to “strongly agree”). Version 1 was therefore a 19-item instrument, with 17 closed and two open-ended questions which were subject to the first phase of the psychometric evaluation.

### Psychometric evaluation

The psychometric evaluation of TEXP-Q involved data from cognitive interviews, content validity indexing and judgement of an expert panel, which together provided information on face and content validity. A subsequent cross-sectional study allowed evaluation of content and factorial (structural) validity, reliability, and responsiveness. To ensure clarity, information is presented as phase I (cognitive interviews, content validity indexing, and expert panel) and phase II (cross-sectional study).

### Phase I

#### Sample and data collection

Cognitive interviews were performed with six emerging adults (five women, one man, all 18–19 years of age), who had made at least three visits to their adult diabetes clinic. In contrast to empirical, qualitative interviews, cognitive interviews play a role during instrument development by shedding light on the items included in the instrument; their formulation, understandability, order and intended focus [17]. Interviews were performed adopting a think-aloud approach, allowing for reflection on the content and formulation of items, thereby revealing respondents’ understanding of the questions, as well as their underlying processes and response selection [18]. As for the content validity indexing, seven diabetes nurses and eight diabetologists at two paediatric and three adult diabetes units (ten women, five men with 1.5–37 years of experience from diabetes care) were asked to rate each item of the instrument. This was done to attain a quantitative measurement of validity in terms of a content validity index (CVI) [18]. Participants were asked to rate each item on a four-point scale from “not relevant” to “highly relevant”, and scores were calculated as the number of respondents agreeing with the item as being “relevant” or “highly relevant”. Finally, an expert panel comprising two patient representatives and the head nurse of one of the major diabetes units in Sweden was asked to review the instrument and recommend final adjustments.

#### Data analysis

During the cognitive interviews, the instrument was assessed both as a whole and on a detail level. Complicated or unclear wording or meaning was carefully documented. Regarding the CVI, scores were assessed at both item level and scale level, with recommended cut-off values of 0.78 for items and 0.90 for scale, respectively [19].

### Phase II

#### Sample and data collection

A cross-sectional study was conducted at eight adult diabetes outpatient clinics in Sweden. Ethical approval was obtained from the Ethical Review Agency in Sweden (Dnr 2020–04679/2022-01370-02). Inclusion criteria were spoken and written Swedish literacy, at least four years’ diabetes duration, and at least two completed visits at the adult diabetes clinic. In Sweden, transfer from paediatric to adult care is performed by the age of 18y. Based on patient lists from patients’ medical charts, all eligible participants were contacted via postal letter, followed by phone call or SMS (n = 357). Letters included study information and a consent form, which responders were asked to return with the inclusion of their email addresses for distribution of the web-based questionnaire. Responders who chose to answer via SMS were asked for their email addresses and could consent to participation at the beginning of the web-based questionnaire. Initial non-responders were sent two separate reminder SMSs before being classified as “non-responder”. Data collection was performed between December 2021 and August 2022 using REDCap, a web-based application for data gathering and storage [20]. For participants who initially consented but did not complete their questionnaire, six e-mail reminders were sent automatically upon absence of response. The final sample included in the analyses consisted of 163 participants – a response rate of 46%. For background characteristics of the study sample, see Table 1. Glucose control in terms of mean HbA1c is 57.3 mmol/mol in the sample is lower compared to 61.5 mmol/mol in the total young T1D population in Sweden 2021 (www.ndr.se).

**Table 1:** Sociodemographic and clinical aspects in 163 emerging adults with type1 diabetes

Age M (SD)*	20.3 (0.9)
Sex n (%)	
Women	79 (48.5)
Men	80 (49.1)
Other	4 (2.5)
Living situation n (%)	
Living alone	30 (18.5)
Co-habitation	30 (18.5)
Living with parent/parents	100 (61.7)
Other	2 (1.2)
Diabetes duration, years, Mdn (min-max)*	11 (4–20)
HbA1c, mmol/mol, M (SD)* / Mdn (min-max)	57.3 (15.3) / 55 (34–130)
Time to follow-up**, days, Mdn (min-max)	68 (0-502)

* M = mean, SD = standard deviation, Mdn = median

** Number of days between last visit at the paediatric diabetes clinic and first visit at the adult diabetes clinic

#### Data analysis

To evaluate the psychometric properties of TEXP-Q an approach based on Classic Test Theory was followed in combination with exploratory factor analysis (EFA) [21]. The number of missing values and invalid scores was calculated as mirroring content validity, since these values and scores are considered an indirect measurement of how intelligible items are [22].

As no subscale structure underlies the development of TEXP-Q, structural validity was investigated using EFA, which allows the identification of dimensions embedded in the instrument idea, explaining item order and structure [23]. Due to ordinal and non-normally distributed data, a polychoric correlation matrix formed the basis of the EFA. Bartlett’s test of sphericity, as well as the Kaiser-Meier-Olkin (KMO) measurement were applied to assure data was factorable. Requirements are a significant Bartlett’s test (p < 0.05) and a KMO statistic exceeding 0.6 [18, 24]. Parallel analysis and a visual scree test were employed to reveal the number of factors to evaluate in factor analysis [23]. Once the number of factors to evaluate was determined, factor analysis with iterated principal axis (PA) was used. PA was preferable given the data is non-normally distributed and small in sample size. PA with initial communalities was performed, allowing the identification of items with factor loadings below 0.4 [25, 26]. Data was rotated using promax (oblique) rotation rather than orthogonal rotation, as it allows emerging of factor intercorrelation [23]. Different factor structures were evaluated based on communalities, as well as an intention to achieve a simple structure defined as no cross-loadings (items that have a factor loading over 0.3 on more than one item [23], a minimum of three items per factor, and acceptable factor loadings value (above 0.4) (23). Additionally, the cumulative variance explained by the chosen factor structure should exceed 60% to be considered adequate [18]. Therefore, several factor analyses were undertaken where two, three or four factors were tested. Additionally, a higher-order factor was evaluated, with the exploratory aim of attempting to understand whether there is a higher factor and to calculate a total scale score.

Internal consistency in terms of Cronbach’s alpha was calculated as a measure of reliability. Values exceeding 0.7 were considered acceptable [18]. Cronbach’s alpha was initially calculated for the complete instrument and was then recalculated for each factor identified through EFA. Finally, responsiveness was specified by floor and ceiling effects and was considered violated if more than 15% of participants received the lowest or highest possible score [27]. Analyses were performed using the psych package in R [28] and IBM SPSS Statistics (version 28).

## Results

### Phase I

During cognitive interviews, respondents reported an overall impression of understandability, appropriateness, and extent. Among the items in the 19-item version (version 2), two received CVI values below the required limit of 0.78 (0.6 and 0.73), while the remaining exceeded this limit. On a scale level, the closed question part of the instrument reached a CVI of 0.92, whereas the open-ended part received 1.0. Combining the results from the cognitive interviews and CVI values, one item was reformulated and moved to the open-ended items. Other items changed regarding order, and/or were reformulated (version 3). Following the expert panel review, the instrument was finally adjusted, with no change in content. However, an alteration of the Likert scale from seven to five points was conducted, based on feedback in the cognitive interviews, ending up in a final test-version (version 4). This test version consisted of 17 questions answered on a five-point Likert scale (“strongly disagree” to “strongly agree”), and three open-ended questions answered in free text, focusing on potentially facilitating aspects of the healthcare transition and transfer.

### Phase II

#### Content validity

No invalid scores were present, and the proportion of missing values ranged from 0.6 to 1.8 (see Table 2.), which together indicate good content validity.

**Table 2:** Missing values and factor loadings after rotation

		Factor loading after rotation		
Item	Missing values n (%)	Factor 1	Factor 2	Factor 3
1. My previous health care provider talked to me in a way that is easy to understand	0	0.170 ^b^		0.791^a^ 0.679 ^b^
2. My previous health care provider listened carefully to me	0			0.887 ^a^ 0.958 ^b^
3. My previous health care provider respected how my customs and beliefs might affect *my diabetes*	2 (1.2)			0.796 ^a^ 0.818 ^b^
4. My previous health care provider and I discussed the realities of being a young person (i.e. thoughts and challenges during this life phase)	0	0.586 ^a^ 0.605 ^b^		0.260 ^a^ 0.180 ^b^
5. My previous health care provider offered me visits at the clinic without my parents/legal guardians	0	0.696 ^a^ 0.780 ^b^	-0.247 ^a^ -0.215 ^b^	
6. I could talk to my previous health care provider about sensitive or difficult issues (even when they had nothing to do with *my diabetes*)	0	0.784 ^a^ 0.821 ^b^	-0.134 ^a^	
7. My previous health care provider and I worked together to develop my ability to autonomously take care of my health and well-being	0	**0.569** ^**a**^ 0.592 ^b^		**0.393** ^**a**^ 0.312 ^b^
8. My previous health care provider and I worked together to develop my ability to autonomously take care of *my diabetes* in everyday life (i.e. medication and self-management)	0	0.354 ^a^		0.577 ^a^
9. My previous health care provider and I worked together to develop my ability to autonomously handle *diabetes-related emergencies*	0	**0.364** ^**a**^	0.115 ^a^	**0.390** ^**a**^
10. My previous health care provider offered me opportunities to meet other young people with *diabetes*	1 (0.6)	0.613 ^a^		
11. My previous health care provider and I worked together to prepare for and plan my future (i.e. education, employment, and relations)	1 (0.6)	0.775 ^a^ 0.695 ^b^	0.142 ^b^	
12. My previous health care provider and I have discussed legal changes to privacy, decision-making and consent that take place at age 18	2 (1.2)	0.633 ^a^ **0.526** ^**b**^	0.301 ^a^ **0.380** ^**b**^	-0.137 ^a^ -0.111
13. My previous health care provider and I worked together to prepare a written transition plan, with health-related goals that arise from my abilities and needs	2 (1.2)	**0.430** ^**a**^ 0.282 ^b^	0.644 ^b^	**0.537** ^**a**^
14. I was involved in the planning of follow-up in adult care by identifying a new adult provider to transfer to	3 (1.8)	0.195 ^a^	0.755 ^a^ 0.912 ^b^	-0.228 ^a^ -0.177 ^b^
15. My previous health care provider involved me in summarizing my time in paediatric care before transfer	2 (1.2)	0.206 ^a^	0.672 ^a^ 0.740 ^b^	
16. When it was time for transfer, I felt prepared to change to an adult health care provider	2 (1.2)	-0.232 ^a^ -0.264 ^b^	0.758 ^a^ 0.682 ^b^	0.278 ^a^ 0.249 ^b^
17. My new adult health care provider was prepared and gave a briefing about my health status and living situation on my first visit	3 (1.8)	-0.288 ^a^	0.655 ^a^	

^a^ EFA with three factors and all items included. Cross-loadings in bold

^b^ EFA with three factors without items 8, 9, 10 and 17. Cross-loadings in bold

Italic: Could be replaced with other chronic conditions

#### Factorial validity

Bartlett’s test of sphericity was significant (*p* < 0.001), and the KMO statistic was 0.856, together indicating that the correlation matrix was factorable and underlying dimensions plausible. PA was applied, producing an initial communalities table wherein item 10 fell behind < 0.4 whereas remaining items exceeded this limit. The unrotated visual scree test, as well as parallel analysis, suggested four factors to be extracted, while only looking at eigenvalues indicated a three-factor model. Thus, both alternatives were further investigated, as was a two-factor solution, aiming at simplicity of the final structure. Communalities after rotation drew attention to items 10 and 17, which did not exceed 0.4. Analyses were therefore repeated both with and without these items, repeatedly producing more adequate factor solutions without them. Every time an item was removed, the factor structure was re-evaluated through parallel analysis and visual scree plot evaluation, which was followed by factor analysis of the suggested factor solution. Once items 10 and 17 were removed, the factor analysis showed that items 7, 8 and 9 consistently cross-loaded. Hence, alternative factor structures were tested in which items 8 and 9 were dropped. The formulation of these three items was similar and item 7 was kept, as it had high communality and was generic, i.e. not specifically focused on diabetes care.

New analyses were undertaken without items 8, 9, 10 and 17, whereupon data suggested a three-factor solution. Nonetheless, a comparison was made between a four, three and two-factor solution to achieve the best structure possible. The number of items with cross-loadings was considerably reduced when applying two or three factors, but for the former, the cumulative variance did not exceed the required limit, so this alternative was rejected. Accordingly, a four-factor solution did not fulfil the requirement of a minimum of three items per factor. Altogether, the three-factor solution was recognised as the most adequate solution, accounting for 60% cumulative variance and a single cross-loading on item 12. This item was carefully discussed amongst the co-authors, and was subsequently rephrased and placed in factor 1, according to its highest loading. After EFA, the instrument comprised 13 questions in total, divided into three latent factors (see Table 3). Following careful reflection on common traits among items connected to each factor, the factors were named. The final names were: “Autonomy and participation”, “Transition and transfer preparation”, and “Healthcare-provider communication”. An evaluation of a higher-order factor showed that while the three factors have adequate factor loadings, the total variance explained by them is low (0.47).

**Table 3:** Final version with three factors and Crohnbach’s alpha values

Factors and items	Reliability *
Autonomy and participation	0.816
4. My previous health care provider and I discussed the realities of being a young person (i.e. thoughts and challenges during this life phase)	
5. My previous healthcare provider offered me visits at the clinic without my parents/legal guardians	
6. I could talk to my previous health care provider about sensitive or difficult issues (even when they had nothing to do with *my diabetes*)	
7. My previous health care provider and I worked together to develop my ability to autonomously take care of my health and well-being	
11. My previous health care provider and I worked together to prepare for and plan my future (i.e. education, employment, and relations)	
12. My previous health care provider and I have discussed legal changes to privacy, decision-making and consent that take place at age 18	
Transition and transfer preparation	0.787
13. My previous health care provider and I worked together to prepare a written transition plan, with health-related goals that arise from my abilities and needs	
14. I was involved in the planning of follow-up in adult care by identifying a new adult provider to transfer to	
15. My previous health care provider involved me in summarizing my time in paediatric care before transfer	
16. When it was time for transfer, I felt prepared to change to an adult healthcare provider	
Healthcare-provider communication	0.799
1. My previous healthcare provider talked to me in a way that is easy to understand	
2. My previous healthcare provider listened carefully to me	
3. My previous health care provider respected how my customs and beliefs might affect *my diabetes*	

*Crohnbach’s alpha

#### Reliability

Initial Cronbach’s alpha for the complete instrument was 0.866, which indicates good internal consistency. Crohnbach’s alpha approximated to 0.8 for all factors respectively (Table 3).

#### Responsiveness

No participant had the highest or lowest possible score, proving no floor or ceiling effects.

## Discussion

### Implications of the findings in the context of existing research

Emerging adults with chronic conditions call for youth-friendly, person-centred healthcare during the transition to adulthood and transfer to adult care [7, 29] yet both research and the supply of validated instruments focusing on emerging adults’ experiences prior to, during, and after transfer is scarce [1, 8]. Although two existing instruments measuring aspects related to transitional care were used as inspiration during the development phase of TEXP-Q [15, 16], these do not specifically apply a person-centred approach, and are both voluminous and thereby more time-consuming in their setup, which might hamper clinical applicability. The aim of this study, therefore, was to develop and psychometrically evaluate TEXP-Q, a new, person-centred questionnaire pinpointing young peoples’ experiences of preparation for their healthcare transition and transfer. Psychometric evaluation resulted in a 13-item, relevant, comprehensive instrument with good validity, reliability, and responsiveness. Through EFA, an underlying factor structure was revealed, dividing items into three latent factors: “Autonomy and participation”, “Transition and transfer preparation”, and “Healthcare-provider communication”.

Recent studies have shown positive relationships between patient-reported experiences and patient-reported outcomes, as well as between patient-reported experiences and external measures of health-care processes and outcomes [13, 30]. As for emerging adults with T1D, Garvey, Foster [31] have shown increased risk of impaired clinical attendance post-transition if they are feeling insufficiently prepared for the transfer to adult diabetes care. Considering patients’ perspectives on their transition and transfer process is consequently important in understanding, and by extension enabling, the inclusion and fusion of components facilitating this process. In parallel, researchers have increasingly proven the positive impact of PCC on various domains, such as quality of life, symptom relief, self-esteem, and health-care utilization and costs [32]. Adopting a person-centred perspective in TEXP-Q is thus well anchored in both the literature and in the adolescent community [29, 33]. Likewise, gathering emerging adults’ own perspectives on their care by using TEXP-Q will hopefully contribute to a better understanding of what content and structure young people call for, and how these requests might be fulfilled and implemented in clinical practice. Hence, it could be used to improve transitional care in various settings.

In versions 1 to 4 of TEXP-Q, all questions but one focused on participants’ previous health-care providers and aspects of the healthcare given pre-transfer. However, one question (question 17) did focus on the reception of emerging adults in adult care post-transfer, but during EFA this was removed due to low communality and loading. On the one hand, this makes theoretical sense and contributes to stringency of the final, psychometrically evaluated instrument suggestion. On the other hand, participants’ experiences of the adult care reception immediately after transfer were consequently lost. More studies are therefore needed to cover these aspects of the transition and transfer processes. It may also be desirable to develop an instrument with the same person-centred approach as TEXP-Q, aiming to capture participants’ experiences post-transfer.

During EFA, factor structure solutions with two, three and four factors were tested. In line with recommendations about honouring simple structure, both the amount of cross-loadings and the cumulative variance were taken into consideration [23]. However, there are different opinions as to how to treat cross-loadings. Some simply recommend either deletion or reformulation of cross-loading items [34], while some express that “complex loadings may not be problematic if there is a clear theoretical reason to believe that the measured variable is influenced by more than one latent construct” (23 p. 235). As mentioned, questions 7, 8 and 9 in this study repeatedly cross-loaded, irrespective of structure solution, which subsequently made sense since they appeared to be too similar in content and formulation. Deletion of questions 8 and 9 and reformulation of question 7 were therefore regarded as most suitable to reduce complexity without losing the specific aspect covered. Regarding the remaining cross-loading of question 12, the content was not covered by any other item, which is why it was considered important to keep in the final version. One could argue that there *was* a theoretical reason to believe it was “influenced by more than one latent construct”, but the decision must nevertheless be taken as to where to locate it. Weighing up its denotation and factor loading, question 12 was finally rephrased and placed in “Autonomy and participation”. Likewise, the deletion rather than reformulation of question 10 was carefully discussed. In contrast to the last-mentioned items, this question was already noticeable due to its low initial communality before rotation. Looking at its content, it could be argued that it might be less relevant to participants at the point of investigation than expected, and that it had less to do with the healthcare given during transition and transfer than expected beforehand. Combining these presumptions with results from the statistical analyses, in which item 10 repeatedly contributed to complexity instead of simplicity, it was finally deleted.

### Methodological considerations

Several strengths of this study ought to be mentioned. Most importantly, it describes the thorough development and evaluation of a new instrument in an area where validated questionnaires are rare. Development included both emerging adults with T1D and healthcare personnel as well as either party experts in their field, thus contributing to relevance of the instrument to both patients and healthcare providers. Analyses covered reliability, responsiveness, and different validity dimensions, all of which proved to be satisfying. Last but not least, the second phase of the evaluation involved the target population, thereby improving the relevance and interpretation possibilities of the results.

However, some limitations need to be highlighted. Firstly, the modesty of the sample size might complicate interpretation of findings. A general rule of thumb in psychometric evaluation is to include 10 times the number of items in the instrument under investigation, which was also the goal in this study. Although close enough, this was not fully achieved, despite active efforts during enrolment. However, it has been previously recommended that studies could include a similar sample size that has worked well in similar studies [35]. Therefore, based on previous evidence, our sample size is acceptable. Secondly, the modest response rate gives rise to a discussion about selection bias, whereby both individual perception of living with T1D and satisfaction with received healthcare may influence the decision to participate or not in the survey. Thirdly, and closely connected to the last-mentioned phenomenon, generalisability of findings must be highlighted. The study group showed a better glycaemic control compared to a comparable national sample. In addition to the selection bias, the fact that only one diagnosis was included which aimed at evaluating a generic instrument may mean the study sample is not representative of the extended group of emerging adults with chronic conditions. Moreover, only five participants were born outside Sweden. Future studies covering other chronic conditions, as well as emerging adults with different countries of birth, are thus required to increase generalisability of both instrument usage and study findings. Fourthly, a higher-order factor was evaluated. However, it is important to acknowledge that it is not standard practice to evaluate a higher-order factor without having confirmed the proposed factor structure through confirmatory factor analysis in a separate dataset [34]. Hence, future studies should focus on confirming the three-factor structure and a potential higher-order factor. Finally, evaluation of some aspects of validity and reliability need longitudinal data and were correspondingly not covered in this study.

## Conclusion

TEXP-Q is a newly developed, person-centred instrument which has proven to be both valid and reliable when applied to youths with T1D. It fills a vital gap in the current instrument selection focusing on emerging adults’ experiences of preparation for transition and transfer. Emerging adults with chronic conditions comprise a vulnerable group, especially during transition to adulthood and transfer to adult healthcare. They also constitute an invaluable source of information and knowledge about facilitators and barriers to the transition and transfer process. Collecting and honouring their personal experiences is therefore of utmost importance in order to make any necessary adjustments to better meet their needs. With this contribution we hope to bridge the gap between research and clinical practice for the benefit of emerging adults with chronic conditions. More studies are needed to include a broader set of diagnoses to aid the generic use of the instrument and to capture the experiences of the reception in adult care.

## Data Availability

Data analysed in the current study are available from the corresponding author on reasonable request.

## Abbreviations

CVI: Content validity index
EFA: Explorative factor analysis
KMO: Kaiser-Meier-Olkin statistic
PA: Principle axis
PCC: Person-centred care
PREMs: Patient-reported experience measures
PROMs: Patient-reported outcome measure
T1D: Type 1 Diabetes
TEXP-Q: Transitional care EXPeriences Questionnaire
